# Oxytocin Increases the Influence of Public Service Advertisements

**DOI:** 10.1371/journal.pone.0056934

**Published:** 2013-02-27

**Authors:** Pei-Ying Lin, Naomi Sparks Grewal, Christophe Morin, Walter D. Johnson, Paul J. Zak

**Affiliations:** 1 Department of Psychology, University of Southern California, Los Angeles, California, United States of America; 2 Department of Psychology, Claremont Graduate University, Claremont, California, United States of America; 3 Fielding Graduate University, Santa Barbara, California, United States of America; 4 Center for Neuroeconomics Studies, Claremont Graduate University, Claremont, California, United States of America; 5 Center for Neuroeconomics Studies, Claremont Graduate University, Claremont, California, United States of America; Centre national de la recherche scientifique, France

## Abstract

This paper presents a neurophysiologic model of effective public service advertisements (PSAs) and reports two experiments that test the model. In Experiment 1, we show that after watching 16 PSAs participants who received oxytocin, compared to those given a placebo, donated to 57% more causes, donated 56% more money, and reported 17% greater concern for those in the ads. In Experiment 2, we measured adrenocorticotropin hormone (ACTH) and oxytocin levels in blood before and after participants watched a PSA. As predicted by the model, donations occurred when participants had increases in both ACTH and oxytocin. Our results indicate that PSAs with social content that cause OT release will be more effective than those that do not. Our results also explain why some individuals do not respond to PSAs.

## Introduction

Advertising seeks to persuade people to purchase a good or service, or to adopt a desired behavior. In 2010, $131 billion were spent in the U.S. to persuade people to engage in some action after viewing, listening to, or reading, an ad [1]. So far, there is no secret formula to designing an effective marketing campaign. There is an old saw in which a marketer says, "Only one-half of my advertising budget is effective; I just don't know which half." That is the marketer's dilemma: how to determine which ads are effective. This paper reports two neuroscience experiments designed to identify why public service ads are effective by measuring--and pharmacologically manipulating--the brain mechanisms that are expected to produce behavioral effects.

We choose to use public service advertisements (PSAs) because they provide a clear metric of behavioral change: donating money to the featured cause. Many PSAs produce attitude changes toward issues in ads, but actions do not always follow positive attitudes. One way to increase attitude strength is to grab participants’ attention [2]. Indeed, most of the empirical marketing literature has focused on the attentional effects of advertising – how successful ads stand out from the thousands of messages consumers see each day [3]–[4]. While the strength of an attitude is associated with behavioral changes [5], why attitude changes do not always lead to actions is a mystery.

In the present study, we adapted the first formal advertising model, AIDA (Attention, Interest, Desire, Action) [6] to identify the physiologic correlates of attention and action in the context of PSAs. In our augmented model, we propose that adrenocorticotropic hormone (ACTH) and oxytocin (OT) are key neurologic contributors to successful PSAs due to their relationships with attention and action. Our mapping from physiologic factors to the AIDA model is imperfect, as we focus solely on attention and action, but it is included for pedagogical purposes. By extending the AIDA model, we ground our approach in an existing framework and generate testable hypotheses that seek to identify why some PSAs, particularly those with social content, are effective.

### Attention

The AIDA model posits that attention, interest, desire, and action are the four components of successful advertisements. The goal of a PSA is to produce a particular behavioral action, and the first step is to attract viewers' attention. Eye-tracking studies have shown, for example, that attention to an ad is related to purchase decisions [7] and that reduced attention can have an adverse impact on sales [8]. For advertisements that last 30 seconds to two minutes, the likely neurochemical signal of attention is ACTH [9]–[12]. ACTH is a mediator of attention and distress [13]–[15]. It is released in seconds following a stimulus [16] and prepares the organism to act in response to environmental change. In animal studies, ACTH inhibits habituation [10] indicating that attention can be sustained in spite of repetition. In humans, ACTH helps maintain performance over time [11] and increases visual attention [14]. Without attention to the ad, the message in the PSA is unlikely to produce an action.

We measure ACTH rather than cortisol because message stimuli (ads) are of limited duration, typically too short for a change in cortisol to occur [17].

### Action

PSAs are designed to persuade people to make positive changes in their lives or to help others [18]. They can raise awareness of potential dangers (such as drug abuse, smoking, or obesity), but may or may not be effective in affecting behavior [19]. Several researchers have shown that effective PSAs elicit empathy in viewers [18], [20]–[21]. For example, Shen (2010) used high and low empathy television PSAs about smoking and drunk driving to show that messages that elicited greater empathy were more effective in changing attitudes [22]. Similarly, televised fund-raising appeals that focused on the benefits to others resulted in larger donation pledges than appeals that focused on the self [20].

Ads that elicit empathy may have a behavioral effect because the ad viewer shares the emotions of the characters shown in the ad. For example, participants who were asked to listen to an interview from a convicted drug user's perspective tended to give more money to a drug-related charity than those directed to listen from their own perspective [23]. Empathy may change behavior because those who are emotionally engaged want to alleviate their own distress [24]–[26], to feel good for supporting a cause [27], or to assuage the suffering of others [28].

Previous studies of messaging effectiveness have used psychological manipulations of empathy [25], [29]–[30]. In these studies, empathy was made salient by instructing subjects to listen to pleas from people requesting assistance, read messages from people asking for help, or to watch people in need on video clips. Participants inevitably respond differently to these stimuli so that the degree of empathic engagement varies and the subsequent impact on behavior is indirect. The neuropeptide oxytocin (OT) has been associated with the subjective experience of empathy in a number of studies. For example, after participants watched a 100 second fund-raising video for St. Jude's Children's Hospital, OT measured in blood increased 47% [31]. In that study, the change in OT correlated with experienced empathy. Barraza and Zak [31] also show that empathy also predicted the money sent to a stranger in a zero-sum monetary transfer.

Studies that infuse synthetic OT into human beings have also documented changes in behaviors associated with emotional engagement, including increased accuracy in recognizing others' emotions from photographs [32], increased charitable giving [33], and an increase in the time spent looking at people's eyes in photographs of faces [34]. A recent study using OT infusion reported an increase in a multifaceted empathy test compared to participants on placebo [35]. This body of research indicates that OT increases empathy physiologically [36]. This literature led us to hypothesize that OT is the neurochemical that motivates action after viewing a PSA.

## A Physiological Model of Effective PSAs

**Figure 1:**
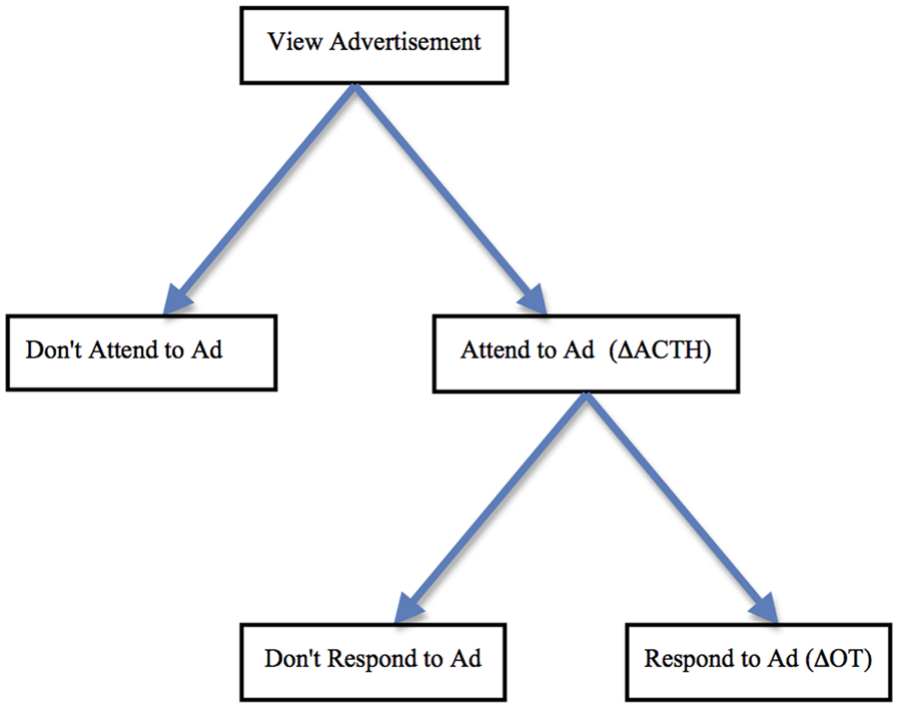
A Physiologic Model of Effective PSAs (PMEP). A physiologic model of attention and action is shown after viewing an advertisement with social content. We propose that attention will be reflected in a sympathetic nervous system response measurable by a change in ACTH. A response to the ad that results in an action will be driven by a change in OT. The model includes cases in which individuals do not attend to the ad and do not respond by engaging in an action after the ad.

The previous section established a relationship between ACTH and attention, as well as between OT and empathy. The Physiologic Model of Effective PSAs (PMEP), presented in Figure 1, adds these two neurochemical correlates to the AIDA model. We hypothesize that when viewing a PSA, attention is quantifiable physiologically by measuring the change in ACTH. Actions following a PSA with social content are hypothesized to be associated with a change in OT. The PMEP posits that it is the ACTH-OT interaction that indicates that a PSA is likely to be effective in both attracting attention and affecting a behavioral change. But why are both necessary?

Physiologically, one must attend to a social stimulus before the release of OT signals the organism to engage in an action [31], [36]. In natural social interactions, the orienting signal can be a sign of distress or a request for help. Advertisements, especially PSAs, often use this strategy, by presenting a scenario in which the characters in the ad are in trouble, or likely to be so. With respect to ACTH and OT release, our previous work suggests that the brain does not effectively differentiate between in-person interactions and those viewed on a computer monitor [31], [37].

The value of the PMEP is that it identifies measurable and objective factors that we hypothesize lead to behavioral actions. Our approach does not rely on self-report attitude changes or hypothetical behaviors. Rather, it is grounded in workings of the brain's autonomic nervous system. Because this part of the brain does not require a conscious decision, most people are unaware that their brains have released ACTH or OT.

If the PMEP is confirmed experimentally, it provides a new approach to evaluate PSA effectiveness for those who create and study these ads. At the same time, there is substantial individual variation in the stimuli that cause, and the amount of, ACTH and OT synthesis so that the PMEP is only expected to hold on average across a population, and not for each particular individual. Further, the PEMP only applies to PSAs and not to advertising relating to goods or services. Purchase decisions have been associated with a change in dopamine in mid-brain regions of the brain that is associated with the desire to obtain a good [38]. In contrast, PSAs nearly always feature social content suggesting a role for OT that may not be found, for example, in an ad for a chocolate bar or new car unless these ads had significant social content.

### Testing the Model

We designed two experiments to test the PMEP. We used PSAs in our experiments because we could measure actual behaviors in response to these ads, donations to the featured charities, rather than simply attitudes. The novel part of the model is the role of OT, so Experiment 1 tests whether OT affects actions in response to PSAs. In Experiment 1, we manipulated OT pharmacologically to establish a causal relationship between OT and donation decisions in response to PSAs. This follows protocols we have used to establish the causal effect of OT on prosocial behaviors between strangers [39]–[40]. If there were no effect of OT on viewers' actions following a PSA, then the proposed model would be invalidated.

It is important to note that OT seldom produces ‘all for you, none for me’ decisions as OT only modulates activity in a network of brain regions that are involved in decision-making [36]–[37], [41]. When the external context and a person's internal state are congruent, OT is likely to have a behavioral effect. This may explain why a PSA may not affect the behavior of all those who hear, read or view it. When a PSA resonates with an individual's personality traits and physiologic state, perceived persuasion is higher and attitude changes have been shown to follow [42]. One of the authors (Morin, C.) provided the PSAs we tested from commercial sources. This does not alter our adherence to PLoS ONE policies on sharing data and materials.

In Experiment 2, we sought to confirm the interactive physiologic mechanisms for attention and action in the PMEP. To do this, we measured endogenous changes in OT and ACTH levels in blood before and after participants watched a PSA from Experiment 1. We expected that the PSA would cause an increase in both ACTH and OT release in most participants. Further, we expected that participants who had increases in both ACTH and OT would be the ones most likely to donate to the charity in the ad.

## Experiment 1: Exogenous OT Infusion

### Methods

#### Ethics statement

The Institutional Review Board at Claremont Graduate University approved this study. No adverse reactions occurred.

#### Participants

A total of 41 healthy male students aged 18 to 32 years old (*M* = 23.2, *SD* = 4.3) were recruited for this study. All participants gave written informed consent prior to the experiment and a lab administrator assigned random numeric codes to participants to mask their identities. The recruiting text solicited participation by noting that participants would earn between $30 and $100. Earnings were paid in cash at the end of the experiment by a lab administrator who was not part of the study to maintain participant anonymity. One participant did not finish the experiment due to computer malfunction, producing a final sample size of 40. Participants' ethnicities were mixed (55% Caucasian, 30% Asian, 12.5%, mixed race, and 2.5% African-American). Women were excluded from the study because the effect of OT varies with a woman's menstrual cycle [43], and because OT contracts the uterus so that there was a possibility that OT infusion could cause an unintended abortion. There was no deception in any part of this experiment.

#### Procedure

After consent, participants were required to pass a medical screening prior to inclusion. Those with serious medical disorders and taking certain medications were excluded from participating because of possible adverse interactions with OT. No exclusions were made. Each participant was randomly assigned to receive either 40 IU of OT (treatment, *N* = 20) or the equivalent volume (4ml) of saline (control, *N* = 20), infused intranasally. The experimenters and participants were blinded regarding the substance each person received.

Participants were then seated at partitioned computer stations in a large lab. They were asked to complete surveys assessing their background, attitudes, and beliefs in the first 10–15 minutes of the 60-minute loading period, following published pharmacokinetics [44] and related studies [40].

After the loading period, participants were instructed that they would view short advertisements on their computer using headphones and be asked about what they saw and how they felt. Participants viewed 16 video clips including audio tracks. The ads were public service announcements from the UK seeking to curtail smoking, drinking, speeding, and global warming. Each ad lasted between 30 and 60 seconds. Ads were counterbalanced across sessions. Immediately after viewing each ad, participants had to report how they felt about the ads (questions below), and were asked to answer a single question regarding the content of the ad. The content questions were designed so that participants could easily answer them. Participants were informed that a correct answer to the content question earned them $5. The post-PSA questions were included to motivate attention to the stimulus and to compensate participants for the experiment.

Following the content question, participants were queried by computer to donate some portion of their $5 earnings to a charity associated with the advertisement. The charities were: Greenpeace, World Wildlife Fund, Mothers Against Drunk Driving, Drug Abuse Resistance Education, Student Against Destructive Decisions, I Have a Dream Foundation, American Cancer Society, and American Lung Association of California. The donation decision was done privately by computer. Donations from participants were sent to the charitable organizations at the end of the study. The 40 participants produced 589 observations of responses to the public service ads they viewed. We excluded 51 responses in which participants gave incorrect answers on the quiz questions. The experiment took approximately two hours to complete.

### Measures

Participants took several personality, behavior, and attitude surveys while the drug loaded to examine potential confounding variables. Surveys included basic demographics, emotional states using ratings of five emotion adjectives (sad, happy, angry, fearful, anxious), the Affective Intensity Measure (AIM; [45]) that assesses emotional stability, and the Interpersonal Reactivity Index and its subscales (IRI, [46]) that measures dispositional empathy. We also obtained data on participants' behaviors and attitudes regarding the four classes of activities in the PSAs (drinking, global warming, smoking, and speeding).

After viewing each PSA, five questions assessed participants' concern for others (other-concern) and concern for one's self (self-concern). The other-concern questions were ‘This ad made me care for the people featured in the ad’; ‘This ad made me care for others I know who are dealing with this particular issue’; and ‘This ad made me want to do something about this issue for others.’ Self-concern was measured by two questions, ‘This ad made me reflect on my own life and how I deal with this particular issue’ and ‘This ad made me want to do something about this issue for myself.’

### Results

#### Main effect

**Figure 2:**
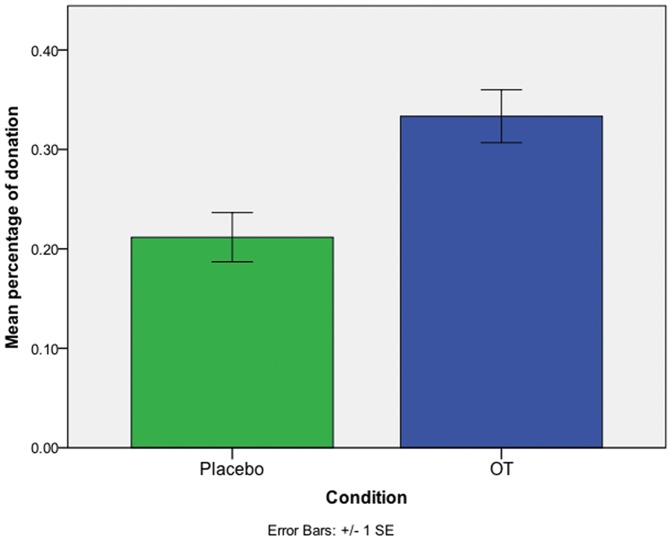
Main effect of OT infusion on percent donated after viewing a PSA. Participants who received OT donated money to 33% of ads for which they correctly answered the content question, while those on placebo donated to 21% of eligible ads ( = 10.835, *p* = .001). The bar represents one standard error. There was no difference between groups in the proportion of correct answers to a question about the ad's content (OT: 93.8%, placebo: 90.1%;  = 2.891, *p* = .091)

**Figure 3:**
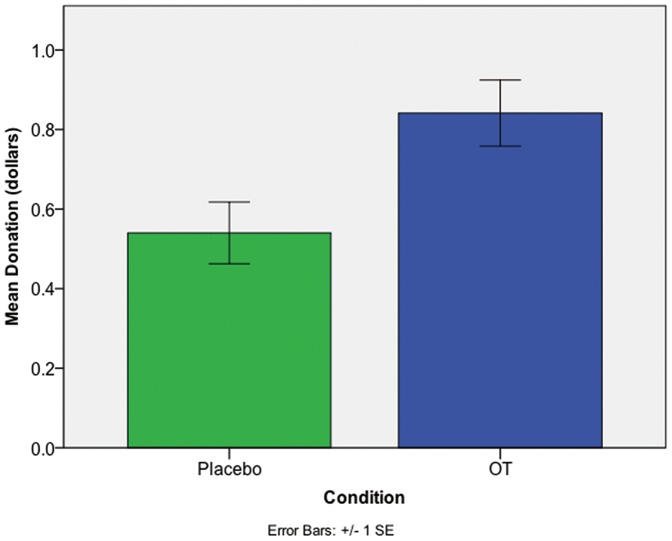
Average dollar donation by condition. Average donations among those who donated to ads +/- one standard error. Those who received OT donated on average 56% more after viewing ads than average donations by those who received a placebo (*p* = .001). This provides behavioral evidence that those on OT were more persuaded by the ads.

Twenty-one percent of the PSAs seen by those on placebo received donations. Participants who received OT made donations to 33% of ads, significantly more than those on placebo (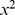 = 10.835, *p* = .001, See Figure 2). Those who received OT donated, on average, 56% more money than those given the placebo (OT: $0.84; Placebo: $0.54; see Figure 3). Since the donation amount was not normally distributed *(Kolmogorov-Smirnov Z* = 1.473, *p* = .03), a non-parametric Mann-Whitney U was performed to test a donation difference across conditions (*p* = .001, two-tailed). The effect of OT remained after controlling for self-concern and other-concern (*t* = 3.59, *p*<.001).

#### Ad content and oxytocin

Next, we investigated whether OT would cause participants to show more concern for the people in the PSAs. The three other-concern questions were highly correlated (*α* = .816) and as a result were averaged into a single measure. The two questions regarding self-concern were also highly correlated (*α* = .869) and were similarly averaged into a single score. Including all participants — those who made donations and those who did not — there was no difference by treatment for other-concern (OT mean: 3.63; Placebo mean: 3.95, two-tailed t-test *p* = .07). Similarly, there was no difference in self-concern across treatments (OT mean: 3.27; Placebo mean: 3.38; two-tailed t-test *p* = .53).

A non-parametric Goodman-Kruskal's gamma test was conducted to test the relationship between donation amount and self/other-concern because the donation distribution was positively skewed due to the high proportion of zero donations. We calculated each individual's gamma for self-concern and for other-concern separately. A positive gamma value for other-concern shows a positive relationship between other-concern and donation amount, and a negative gamma indicates the opposite. Participants were excluded from the non-parametric analysis if they donated nothing or if they donated the same amount to every ad. The distribution of participants included (OT = 21, Placebo = 9) relative to those excluded (OT = 7, Placebo = 10) was not statistically significantly different (*p* = .22). Responses from 23 participants were analyzed for testing the interaction between self/other concern and OT.

**Figure 4:**
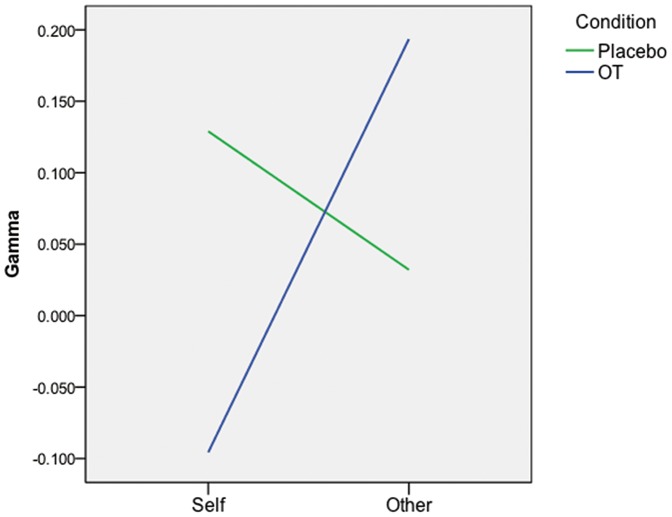
Concern for self and others by condition. When making donations, OT increased concern for others and decreased the concern for self (*p* = .03). This shows that OT selectively affects the persuasiveness of ads that resonant with viewers' physiologic states.

Kolmogorov-Smirnov tests of normality showed that the distributions of gamma values for self-concern and other-concern were not normal (*p*<.001); therefore, a re-sampling procedure was conducted to analyze the interaction effect between OT and self/other concerns. Data were randomly permutated ten thousand times, and each time a 2×2 ANOVA was conducted. Based on the empirical distribution of ten thousand F-values, we found a significant interaction effect between OT and self/other concern (*p* = .03, empirical *F*(1, 21) = 5.28, adjusted η  = .34). Those on OT had a positive relationship (average gamma = .29) between concern for others and donation amount whereas the relationship was minimal for those on placebo (average gamma  = .07). In contrast, those on OT showed a negative relationship between donation amount and self-concern (average gamma =  −0.14) but the relationship was positive in the placebo group (average gamma = .27, see Figure 4). That is, participants who received OT donated more when advertisements elicited concern for others, whereas they donated less when advertisements elicited concern for self. This relationship was reversed for the control group.

Donations were also associated with the mood induced by viewing the ad, and there were distinct differences in the effects of mood between those in the OT and placebo groups. Participants who received OT were more likely to donate when the ads provoked sad, angry, and fearful moods, and when they reported greater trust in the cause (gamma test, *p*<.05; Table 1). For those on placebo, ads that produced both a happy mood and a negative mood received significantly more donations (gamma test, *p*<.05). That different moods affect the OT and placebo groups indicates that OT infusion changed affective state.

**Table 1:** Donation amount and emotion triggered by ads.

		Positive	Negative	Sad	Angry	Fearful	Anxious	Trust	Happy
Oxytocin	gamma	−.110	.184	.221*	.314*	.185*	.127	.175*	.018
Placebo	gamma	−.025	.183*	.176	.192	.107	.082	−.022	.193*

A positive gamma indicates a positive relationship between donation amount and an emotion, while a negative gamma shows the opposite. For those on OT, donation amount was positively related to sadness, anger, fear, and trust. For those on placebo, donation amount was only positively related to happiness and negative emotion. The * denotes a difference between treatments at p≤.05.

Testing for additional effects, we found that ads without much movement by the actors produced more donations for those on OT (Mann-Whitney U test, *p* = .001, *M_still_* = $ 1.08, *M_movement_* = $.51), but not more donations for those on placebo (Mann-Whitney U test, *p* = .63). Ads showing fewer young people received marginally more donations from those on OT (Mann-Whitney U test, *p* = .054,  = $.94, $.59) but did not attract more donations from placebo participants. There was no relationship between OT and donations for ads that featured many versus few people, music or no music, different types of music, ads showing people having fun, ads with threats, ads that varied by gender, or ads that focused on death (*p*s>.05).

There was no significant difference between participants on OT and placebo in the number of close friends (*p* = .71), trait empathy (*p* = .17), satisfaction with life (*p* = .96), and mood prior to viewing ads (*p* = .30). We also tested whether participants' drinking (*p* = .33), smoking (*p* = .19), speeding (*p* = .69), or action against global warming (*p* = .16) differed significantly among participants on OT and placebo, and they did not.

### Discussion

In Experiment 1, we sought to determine whether OT, which enhances empathy and trust, increased donations for PSAs. Those who received OT donated to more causes after viewing ads and had larger average donations than those who received a placebo. Our analysis indicated that OT affected donations by selectively altering the susceptibility to concern for self and others, moving participants to act in response to ads that induced concern for others. It should be noted that OT did not increase concern for people in all ads, but when OT elicited greater concern, donations followed.

Participants who received a placebo were persuaded to donate by ads with greater self-relevance, while those on OT were more persuaded by ads that concerned other people, consistent with OT's ability to alter the self-other balance [36]. This result also reveals the importance of matching personal states with advertisement content. Although participants on OT did not report greater concern for all ads, they reported more engagement and opened their wallets when the ad content was congruent with their heightened empathy. This suggests that a PSA that raises OT will be most effective when it directs individuals' attention to the needs of others. Absent such an approach, ads focused on the viewers' own needs appear to be most effective.

While it is important to acknowledge that many factors affect decisions to donate to charities other than the ones studied here, our results complement previous findings from studies of OT infusion and the sharing of money with a stranger. In one study, intranasal infusion of 24IU of OT caused a 17% larger monetary transfer denoting trust in an unknown person in the lab compared to those given a placebo [39]. Monetary transfers were made in this experiment because of the expectation of a larger return of money from the person who was trusted. What is surprising in the present study is that the alteration in the self-other balance occurred when participants watched public service ads with actors portraying fictional scenes and that this caused out-of-pocket donations to the issues promoted in the ads. This finding complements the increase in generosity to another person after an infusion of 40IU of OT in a zero-sum setting [40] and in donations to charity [33].

The affective mechanism at work appears to be enhanced empathy. Previous findings have associated endogenous OT release, as well as exogenous OT infusion, with increased empathy [31], [35]. Empathy has also been shown to boost the persuasiveness of advertising [18]. The research reported here is the first to show increased advertising effectiveness when empathy was raised in a physiologically consistent way across participants. Interestingly, our findings were not associated with an individual's trait empathy as measured by the IRI. This is important because trait empathy has been positively associated with greater endogenous OT release when viewing a nonfictional emotional video [31].

## Experiment 2: Testing the Full Model

Experiment 1 demonstrated that exogenous OT increases actions in response to PSAs. Experiment 2 was designed to test both the attention and action parts of the PMEP by measuring endogenous changes in ACTH and OT. We selected a single ad from Experiment 1 to see if viewing it would cause the brain to synthesize and release ACTH and OT in healthy male and female participants. OT infusion is known to carry a small risk of miscarriage for pregnant females, and therefore most OT infusion studies, including our Experiment 1, exclude women. Male and female participants were included in Experiment 2 as there was no reason for a gender exclusion when measuring endogenous OT release. The PMEP predicts that when an individual has an increase in both ACTH and OT, donations will be larger than in participants who lacked one or both of these physiologic effects.

### Methods

#### Ethics statement

The Institutional Review Board at Claremont Graduate University approved this study. There were no adverse reactions to the study.

#### Participants and procedures

We recruited 42 healthy male and female students aged 18 to 35 years old (*M* = 21.0, *SD* = 3.2) for this experiment. Those who participated in the first experiment were not permitted to join the second experiment. Participants gave written informed consent prior to inclusion and a lab administrator assigned random numeric codes to mask participants' identities. As in Experiment 1, ethnicities were mixed in Experiment 2 (55% Caucasian, 16.7% Asian, 11.7% other, 9.5% African-American, and 7.1% Hispanic). There was no deception in any part of this experiment. On average, participants earned $38.10 (*SD* = 3.36). Four participants were excluded from analyses because their OT levels were outside of the acceptable assay range (>2500 pg/ml). The final sample size was 38.

After consent, participants were led to a private room for their first blood draw by a licensed phlebotomist. Participants were then seated at partitioned computer stations and asked to fill out a survey about basic demographics, current moods, and empathic concern (IRI). Once all participants finished the survey, participants viewed a brief advertisement (59 seconds) about smoking, and were asked to fill out a survey rating how strongly they felt particular emotions. No interpersonal communication was permitted among participants. Immediately following the second survey, a second blood draw was performed. Participants were then asked to answer a question about the content of the ad (all participants answered it correctly). Participants received $40 after correctly answering the content question and decided how much to donate to an anti-smoking charity. When all tasks were complete, participants were privately paid by a lab administrator who was not associated with the experiment.

### Measures

#### Blood draws

After consent, all participants had 20 ml of blood drawn from an antecubital vein. Two 6 ml EDTA whole-blood tubes and one 8 ml serum-separator tube were drawn while maintaining a sterile field using a Vacutainer blood draw kit (BD, Franklin Lakes, NJ, USA). The second 20 ml blood draw was done within two minutes after viewing an ad.

Blood tubes were immediately placed on ice after being drawn. The tubes were then placed in a refrigerated centrifuge and spun at 1500 rpm for 12 min at 4°C. Plasma and serum were aliquoted from the tubes and placed into 2 ml microtubes with screw caps. These tubes were immediately placed on dry ice and then transferred to a −80° C freezer until analysis.

#### Assays

Two hormones were assayed to measure attention and action as in the PMEP, ACTH and OT. ACTH was assayed using radioimmunoassay (RIA) using a kit produced by DiaSorin, Inc. (Stillwater, MN, USA), and OT was assayed using enzyme-linked immunosorbent assay (ELISA) using a kit produced by ENZO Life Sciences (Farmingdale, NY, USA). The inter- and intra-assay coefficients of variations for ACTH were 8.1% at 75.3 pg/ml and 5.8% at 52.0 pg/ml, and the values for OT were 7.5% at 484.7 pg/ml and 10.2% at 494.6 pg/ml using 10 replicates for both assays. Both are within acceptable ranges. All tests were performed by the Biomarkers Core at the Yerkes National Primate Research Center in Emory University, Atlanta, GA.

#### Advertisement

All participants, using headphones, privately viewed the same 59-second UK television public service advertisement in partitioned computer stations. The advertisement sought to persuade people to stop smoking, and included scenes of adults smoking cigarettes with graphic depictions of clogged arteries. This advertisement was chosen because it elicited the greatest ‘concern for others’ among those in the placebo group in Experiment 1 (*M* = 5.0, *SD* = 2.3).

#### Advertisement ratings

At the end of the advertisement, participants were asked to rate the degree to which they experienced particular emotions while viewing the ad. This list included 12 adjectives used in Experiment 1 to assess empathy toward others (e.g., sympathetic, compassion, moved, tender, warm, soft-hearted; *α* = .82), attention (e.g. mind-wandering), and personal distress (e.g., anxious, distressed, sad, annoyed, frightened, disturbed; *α* = .70). Participants rated these adjectives from 1 (did not feel this way at all) to 5 (felt this way very much).

#### Donation task

After the second blood draw, participants were informed of their study earnings in private and presented with the opportunity to donate any amount of their study earnings to a well-known anti-smoking charity (the American Cancer Society). The experimenters informed participants that there was no obligation to donate and that their decision to donate was anonymous.

### Results

Watching the anti-smoking PSA produced a significant increase in ACTH (*M1* =  52.5 pg/ml, *M2* = 59.1 pg/ml; two-tailed t-test, *p* = .01), indicating that the ad attracted most viewers' attention. As predicted by the PMEP, the change in ACTH was positively correlated to attention to the ad (*r* = .38 *p* = .02). There was no significant overall change in OT levels from viewing the PSA (*M1* = 630.3 pg/ml, *M2* = 627.8 pg/ml; *p* = .94). The changes in ACTH and OT were uncorrelated (*r* =  −.23, *p* = .16). Additionally, basal OT was not related to basal ACTH (*p* = .26).

**Figure 5:**
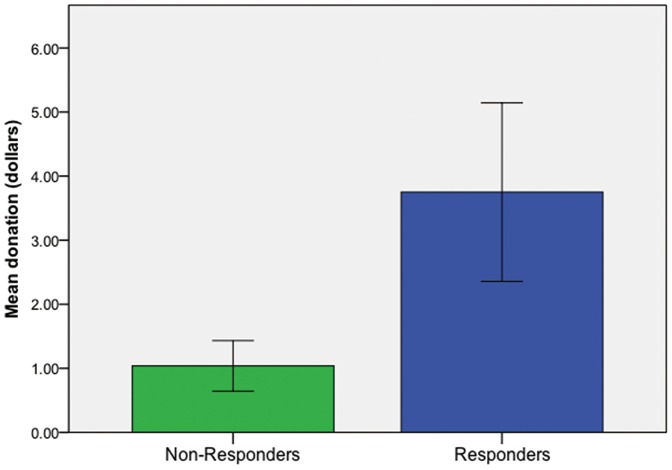
Donations after endogenous responses to PSAs by condition. Responders, those who had an endogenous increase in both ACTH and OT, made donations that were 261% larger than Non-Responders (*p* = .02). This confirms a key aspect of the PMEP model in which action is contingent on attention.

Overall, the change in OT was, in isolation, unrelated to the donation amount (*p*>.05). This was also true for donations and the change in ACTH (*p*>.05). But, as predicted by the PMEP, when we compared participants who had an increase in both OT and ACTH after watching the ad (Responders, *N* = 12) to those whose did not have both effects (Non-Responders, *N* = 26), Responders donated 261% ($2.70) more than Non-Responders (*M_Responders_* = 3.75, *M_Non-Responders_* = 1.04; two-tailed t-test *p* = .02; Figure 5). Both attention and engagement with the ad's characters appear necessary to result in a donation. There were no significant relationships between any mood, personality traits, or smoking behaviors from survey measures and donation decisions (*ps*>.05).

### Discussion

Experiment 2 demonstrated that when a PSA caused an increase ACTH and OT in participants, participants were engaged with the PSA and a behavioral action (donation) followed. This result is consistent with the PMEP model, and complements findings from Barraza and Zak [31] who first documented that a change in endogenous OT after viewing an emotional video was associated with generosity towards a stranger, as well as Barraza, McCullough, & Zak [33] who showed that an exogenous increase in OT caused greater donations to charity without having viewed an ad. While the present contribution advances the scientific understanding of effective PSAs, we acknowledge it does not exhaust all possible ways the AIDA model can be measured or influenced biologically. In particular, we focus exclusively on measuring attention and action as factors that can be directly measured and manipulated experimentally. Interestingly, we did not find an interaction between attention and concern for others based on self-report data (*p*>.05), while it was apparent in the hormone data. This difference further reinforces the value of obtaining physiologic measurements rather than self-reports. The AIDA model is typically tested using self-reports and the findings here suggest this may be inappropriate because the brain mechanisms producing attention and action identified in the PMEP occur largely outside of one's conscious awareness. Indeed, people often are unable and inconsistent in articulating why they are doing what they are doing [47]–[52].

It is important to note that OT alone may increase attention to social cues [53]. In Experiment 2, unlike what we found in Experiment 1, OT alone was not related to donations. This may be due differences in the amount of OT between experiments. In Experiment 1, exogenous OT was used to establish its causal relationship to donations. A study using 40IU of inhaled arginine vasopressin, a peptide only differing in two amino acids from OT, showed an over 700% increase in levels in blood serum (and a 400% increase in cerebral spinal fluid) after an 80 minute loading period [44]. In Experiment 2, Responders had a 19% increase in OT in blood, suggesting the impact of endogenous release on actions following PSAs is smaller and more nuanced than the effect of exogenous OT.

## Applications and Extensions

Advertisers obviously cannot spray OT during viewings of their PSAs, but the findings here, coupled with studies identifying the variety of stimuli that induce OT release, suggest several ways that PSAs and perhaps other marketing efforts that include social content can be made more effective. Indeed, our findings indicate that the brain may not distinguish triggers for OT release that occur in-person compared to those that are viewed through visual media. Activities that induce the brain to release OT include watching an emotional video clip [31], being trusted [41], [54]), being touched [55], attending a wedding [56], petting one's dog [57], moderate stress [58], holding one's infant [59], breastfeeding [60], sexual activity [61], and perhaps even Tweeting [37], [62]. Our findings suggest that advertisements that feature these activities may induce OT release. If our results generalize beyond PSAs, then a body lotion commercial would be more effective when it shows the lotion being spread on someone's skin, especially by another person, than simply featuring the lotion bottle.

Positive in-person interactions also induce OT release [37] and marketing in stores and at events can also be crafted based on the research reported here. If additional research shows that the PMEP extends to ads for products, then in-person marketing should focus on i) attracting attention, and ii) building emotional connections with potential customers that take their needs into account. This approach indicates that effective marketing campaigns should be seen as ways to build relationships and solve customers' problems rather than focusing on a one-time sale. Marketing that causes OT release is a step toward building an emotional relationship with a product or brand.

An important caveat from this research is that those with pharmacologically-enhanced OT in Experiment 1 did not donate to the charity for every advertisement they viewed. The ads that caused participants to donate money varied across individuals. This suggests a ‘one size fits all’ approach to stimulate OT release will not work for advertisers. For example, we have found in every study in our lab that women release more OT after a stimulus than do men [37]. We have also shown that pharmacologically-elevated testosterone, which inhibits the action of OT, decreases generosity toward a stranger in a ‘share the money’ task with a stranger [63]. The OT triggers cataloged above will vary across population segments including gender and other factors so marketers will have to continue to segment the population they seek to influence. While we have unmasked part of the neurobiology of effective public service advertisements, crafting persuasive ads will still require skill and nuance.

## References

[1] Kantar Media reports U.S. advertising expenditures increased 6.5 percent in 2010. Available: http://kantarmediana.com/intelligence/press/us-advertising-expenditures-increased-65-percent-2010. Accessed March 17 2011.

[2] Petty RE, Cacioppo JT (1986) The elaboration likelihood model of persuasion. In L. Berkowitz (Ed.), Advances in experimental social. New York: Academic Press. psychology pp . 123–203.

[3] Milosavljevic M, Cerf M (2008) First Attention then Intention: Insights from Computational Neuroscience of Vision. International Journal of Advertising 27: 381–398.

[4] Sacharin K (2000) Attention! How to Interrupt, Yell, Whisper & Touch Customers. New York: John Wiley & Sons, Inc.

[5] Ajzen I, Cote NG (2008) Attitudes and the prediction of behavior. In W.D. Crano andR. Preslin (Eds.). Attitudes and Attitude Change. New York: Psychology Press.

[6] Strong EKJR (1925) Theories of selling. Journal of Applied Psychology 9: 75–86.

[7] Lohse GL (1997) Consumer eye movement patterns on Yellow Pages advertising. Journal of Advertising 26: 61–73.

[8] Janiszewski C (1998) The influence of display characteristics on visual exploratory search behavior. Journal of Consumer Research 25: 290–301.

[9] Born J, Bathelt B, Pietrowsky R, Pauschinger P, Fehm HL (1990) Influences of peripheral adrenocorticotropin 1–39 (ACTH) and human corticotropin releasing hormone (h-CRH) on human auditory evoked potentials (AEP). Psychopharmacology 101: 34–38. 2160665 10.1007/BF02253714

[10] File SE (1978) ACTH, but not corticosterone, impairs habituation and reduces exploration. Pharmacology Biochemistry and Behavior 9: 161–166. 10.1016/0091-3057(78)90159-4 213788

[11] Gaillard AWK, Varey CA (1979) Some effects of an ACTH 4–9 analog (ORG 2766) on human performance. Physiology and Behavior 23: 78–84. 10.1016/0031-9384(79)90126-4 229501

[12] Spruijt B (1992) An ACTH4-9 analog enhances social attention in aging rats: a longitudinal study. Neurobiology of Aging 13: 153–158. 1311802 10.1016/0197-4580(92)90023-q

[13] Mauri A, Volpe A (1994) Stress mediators in the amniotic compartment in relation to the degree of fetal distress. Fetal Diagnosis & Therapy 9: 300–305. 7818778 10.1159/000263952

[14] Sandman CA, George JM, Nolan JD, van Riezen H, Kastin AJ (1975) Enhancement of attention in man with ACTH/MSH 4–10. Physiology & behavior 15: 427–431 Retrieved from http://www.ncbi.nlm.nih.gov/pubmed/176674. 176674 10.1016/0031-9384(75)90209-7

[15] Sandman CA, George J, McCanne TR, Nolan JD, Kaswan J, et al (1977) MSH/ACTH 4–10 influences behavioral and physiological measures of attention. Journal of Clinical Endocrinology & Metabolism 44: 884–891. 192754 10.1210/jcem-44-5-884

[16] De Weid D, Weijnen JAMW (1970) Progress in Brain Research 32: Pituitary, adrenal and the brain. Amsterdam: Elsevier

[17] Nicholson N (2008) Measurement of cortisol. In L. Luecken, & L. Gallo (Eds.), Handbook of physiological research methods in health psychology. Thousand Oaks, CA: SAGE Publications, Inc. pp. 37–75

[18] Bagozzi DJ, Moore RP (1994) Public service advertisements: Emotions and empathy guide prosocial behavior. Journal of Marketing 58: 56–70.

[19] Murry JP Jr, Stam A, Lastovicka JL (1996) Paid- versus donated-media strategies for public service announcement campaigns. Public Opinion Quarterly 60: 1–29.

[20] Fisher RJ, Vandenbosch M, Antia K (2008) The effects of content, placement, and delivery characteristics on televised fundraising for nonprofit organizations. Journal of Consumer Research 35: 519–531.

[21] Shelton ML, Rogers RW (1981) Fear-arousing and empathy-arousing appeals to help: The pathos of persuasion. Journal of Applied Social Psychology 11: 366–378.

[22] Shen L (2010) Mitigating psychological reactance: The role of message-induced empathy in persuasion. Human Communication 36: 397–422.

[23] Batson CD (2002) Addressing the altruism question experimentally. In: Post SG, Underwood LG, Schloss JP WB, editors. Altruism and Altruistic Love. Oxford: Oxford University Press.

[24] Hoffman ML (1982) Development of prosocial motivation: Empathy and guilt. In N. Eisenberg-Berg, (Ed.), Development of Prosocial Behavior. New York: Academic Press. pp. 281–313.

[25] Batson CD, Fultz J, Schoenrade PA (1987) Distress and empathy: Two qualitatively distinct vicarious emotions with different motivational consequences. Journal of Personality 55: 19–39. 3572705 10.1111/j.1467-6494.1987.tb00426.x

[26] Barraza JA, Zak PJ (2012) Oxytocin: Prosocial emotions and behavior. In E. Choleris, D. Pfaff, andDr M. Kavaliers (Eds.), Oxytocin, Vasopressin and Related Peptides in the Regulation of Behavior, in press, Cambridge: Cambridge University Press .

[27] Arnett DB, German SD, Hunt SD (2003) The identity salience model of relationship marketing success: The case of nonprofit marketing. Journal of Marketing 67: 89–105.

[28] Batson CD (1990) How social an animal? The human capacity for caring. American Psychologist 45: 336–346.

[29] Batson CD, Sager K, Garst E, Kang M, Rubchinsky K, et al (1997) Is empathy-induced helping due to self-other merging? Journal of Personality and Social Psychology 73: 495–509.

[30] Loggia ML, Mogil JS, Bushnell MC (2008) Empathy hurts: compassion for another increases both sensory and affective components of pain perception. Pain 136: 168–176. 17822850 10.1016/j.pain.2007.07.017

[31] Barraza JA, Zak PJ (2009) Empathy toward strangers triggers oxytocin release and subsequent generosity. Annals of the New York Academy of Sciences 1167: 182–189. 19580564 10.1111/j.1749-6632.2009.04504.x

[32] Domes G, Heinrichs M, Michel A, Berger C, Herpertz SC (2007) Oxytocin improves ‘mind-reading’ in humans. Biological Psychiatry 61: 731–733. 17137561 10.1016/j.biopsych.2006.07.015

[33] Barraza JA, McCullough ME, Zak PJ (2011) Oxytocin infusion increases charitable donations regardless of monetary resources. Hormones and Behavior 60: 148–151. 21596046 10.1016/j.yhbeh.2011.04.008

[34] Guastella AJ, Mitchell PB, Dadds MR (2008) Oxytocin increases gaze to the eye region of human faces. Biological Psychiatry 63: 3–5. 17888410 10.1016/j.biopsych.2007.06.026

[35] Hurlemann R, Patin A, Oezguer P, Onur OA, Cohen MX, et al (2010) Oxytocin enhances amygdala-dependent, socially reinforced learning and emotional empathy in humans. Journal of Neuroscience 30: 4999–5007. 20371820 10.1523/JNEUROSCI.5538-09.2010 PMC6632777

[36] Zak PJ (2011) Moral Markets. Journal of Economic Behavior & Organization 77: 212–233.

[37] Zak PJ (2012) The Moral Molecule: The Source of Love and Prosperity. New York: Dutton . PMC3574777 23447796

[38] Knutson B, Rick S, Wimmer E, Prelec D, Loewenstein G (2007) Neural predictors of purchases. Neuron 53: 147–156. 17196537 10.1016/j.neuron.2006.11.010 PMC1876732

[39] Kosfeld M, Heinrichs M, Zak PJ, Fischbacher U, Fehr E (2005) Oxytocin increases trust in humans. Nature 435: 673–676. 15931222 10.1038/nature03701

[40] Zak PJ, Stanton AA, Ahmadi S (2007) Oxytocin increases generosity in humans. PLoS ONE 2 11:e1128 doi:10.1371/journal.pone.0001128. 17987115 10.1371/journal.pone.0001128 PMC2040517

[41] Zak PJ (2004) Neuroeconomics. Philosophical Transactions of the Royal Society B (Biology) 359: 1737–1748. 10.1098/rstb.2004.1544 PMC1693452 15590614

[42] Cesario J, Grant H, Higgins ET (2004) Regulatory fit and persuasion: Transfer from "feeling right.". Journal of Personality and Social Psychology 86: 388–404. 15008644 10.1037/0022-3514.86.3.388

[43] Salonia A, Nappi RE, Pontillo M, Daverio R, Smeraldi A, et al (2005) Menstrual cycle-related changes in plasma oxytocin are relevant to normal sexual function in healthy women. Hormones and Behavior 47: 164–169. 15664019 10.1016/j.yhbeh.2004.10.002

[44] Born J, Lange T, Kern W, McGregor GP, Bickel U, et al (2002) Sniffing neuropeptides: a transnasal approach to the human brain. Nature Neuroscience 5: 514–516. 11992114 10.1038/nn849

[45] Larsen RJ, Diener E (1987) Affect intensity as an individual difference characteristic: A review. Journal of Research in Personality 21: 1–39.

[46] Davis MH (1980) A multidimensional approach to individual differences in empathy. Catalog of Selected Documents in Psychology 10: 85.

[47] Campbell DT (1963) Social attitudes and other acquired behavioral dispositions. In S. Koch (Ed.), Psychology: A study of a science. New York: McGraw-Hill.

[48] Smith RE, Swinyard WR (1983) Attitude-behavior consistency: The impact of product trial versus advertising. Journal of Marketing Research 20: 257–267.

[49] Kaiser FG (1998) A general measure of ecological behavior. Journal of Applied Social Psychology 28: 395–422.

[50] Kaiser FG, Oerke B, Bogne FX (2007) Behavior-based environmental attitude: Development of an instrument for adolescents. Journal of Environmental Psychology 27: 242–251.

[51] Kaiser FG, Wilson M (2004) Goal-directed conservation behavior: The specific composition of a general performance. Personality and Individual Differences 36: 1531–1544.

[52] Nisbett R, Wilson T (1977) Telling more than we can know: Verbal reports on mental processes. Psychological Review 84: 231–259.

[53] Reddon A, O′Connor C, March-Rollo S, Balshine S (2012) Effects of isotocin on social responses in a cooperatively breeding fish. Animal Behaviour 84: 753–760.

[54] Zak PJ, Kurzban R, Matzner WT (2005) Oxytocin is associated with human trustworthiness. Hormones and Behavior 48: 522–527. 16109416 10.1016/j.yhbeh.2005.07.009

[55] Morhenn VB, Park JW, Piper E, Zak PJ (2008) Monetary sacrifice among strangers is mediated by endogenous oxytocin release after physical contact. Evolution and Human Behavior 29: 375–383.

[56] Geddes L (2010) My big fat Greek wedding: Tears, joy, and oxytocin. New Scientist, Retrieved 12-13-11 fromhttp://www.newscientist.com/article/mg20527471.000-my-big-fat-geek-wedding-tears-joy-and-oxytocin.html.

[57] Odendaal J, Meintjes R (2003) Neurophysiological correlates of affiliative behaviour between humans and dogs. The Veterinary Journal 165: 296–301. 12672376 10.1016/s1090-0233(02)00237-x

[58] Heinrichs M, Baumgartner T, Kirschbaum C, Ehlert U (2003) Social support and oxytocin interact to suppress cortisol and subjective responses to psychosocial stress. Biological Psychiatry 54: 1389–1398. 14675803 10.1016/s0006-3223(03)00465-7

[59] Feldman R, Gordon I, Schneiderman I, Weisman O, Zagoory-Sharon O (2010) Natural variations in maternal and paternal care are associated with systematic changes in oxytocin following parent-infant contact. Psychoneuroendocrinology 35: 1133–1411. 20153585 10.1016/j.psyneuen.2010.01.013

[60] Matthiesen AS, Ransjo-Arvidson AB, Nissen E, Uvnas-Moberg K (2001) Postpartum maternal oxytocin release by newborns: effects of infant hand massage and sucking. Birth 28: 13–19. 11264623 10.1046/j.1523-536x.2001.00013.x

[61] Donaldson ZR, Young LB (2008) Oxytocin, vasopressin, and the neurogenetics of sociality. Science 322: 900–904. 18988842 10.1126/science.1158668

[62] Penenberg A (2011) Digital Oxytocin: How Trust Keeps Facebook, Twitter Humming. Fast Company Available: http://www.fastcompany.com/1767125/digital-oxytocin. Accessed July 20 2011.

[63] Zak PJ, Kurzban R, Ahmadi S, Swerdloff RS, Park J, et al (2009) Testosterone administration decreases generosity in the ultimatum game. PLoS ONE 4 12:e8330 doi:10.1371/journal.pone.0008330. 20016825 10.1371/journal.pone.0008330 PMC2789942

